# The importance of depression during pregnancy

**DOI:** 10.5935/1518-0557.20200010

**Published:** 2020

**Authors:** Atefeh Ghanbari Khanghah, Zahra Bostani Khalesi, Rad Hassanzadeh Afagh

**Affiliations:** 1 Social Determinants of Health Research Center, School of Nursing and Midwifery, Guilan University of Medical Sciences, Rasht, Iran; 2 Pediatric Diseases Research Center, Guilan University of Medical Sciences, Rasht, Iran

**Keywords:** depression, pregnancy, outcome

## Abstract

**Objective:**

Today, the high prevalence of depression is one of the major health problems of pregnant women. This study aimed to assess the relationship between antenatal depression, pregnancy and neonatal outcomes.

**Methods:**

This cohort study involved 394 pregnant women referred to the prenatal care clinic at the Al- Zahra Hospital, affiliated to Guilan University of Medical Sciences, Iran. We used a convenient method for sampling. We collected data using questionnaires on demographic and obstetric characteristics, the CES-D scale for depression evaluation and a checklist for recording pregnancy outcomes.

**Results:**

According to the results, preeclampsia, premature membrane rupture, preterm delivery, cesarean section, intrauterine fetal death, and intrauterine fetal growth restriction were higher among mothers with depression during their pregnancies, compared to those who did not have depression. In addition, the mean birth weights of depressed mothers' infants were lower than the infants of mothers who did not have depression.

**Conclusion:**

Results from the present study showed that depression during pregnancy is associated with pregnancy and neonatal outcomes. Healthcare planners and mental health counselors should regard screening mothers with risk of depression and following them up and, in the necessary cases, referring to psychiatrists.

## INTRODUCTION

Depression occurs in approximately one in every five pregnancies, and has been found to occur more often in the prenatal period than in postpartum (Accortt *et al*., 2015). According to reports from the World Health Organization, depression is the fourth reason for disability across the world, and by 2020, it would climb up to the second place (Pearlstein, 2015). Women are more prone to develop depression during pregnancy than at other times in their lives, likely because of their altered hormonal state (Venkatesh *et al*., 2016). Szegda *et al*. (2014) reported that about 4-29% of women would suffer from depression during their pregnancies.

The prevalence of major antenatal depression can vary, and ranges between 10% and 19%, depending on the population studied and the pregnancy stage (Loomans *et al*., 2013). For example, Lee *et al*. (2007) demonstrated that depression was higher in the first and third trimester, while Bowen & Muhajarine (2006) found that socially high-risk participants had more symptoms of depression than participants who were not socially at high-risk. Research demonstrates that unplanned pregnancy can significantly increase a woman’s likelihood of developing antenatal depression (Pearlstein, 2015; Loomans *et al*., 2013; O'Mahen *et al*., 2013). Bunevicius *et al*. (2009) conducted a longitudinal study with 230 participants, screening them for depression at three trimesters. At all three trimesters, a history of depression and an unwanted or unplanned pregnancy significantly predicted the likelihood of antenatal depression.

Generally, risk factors such as personal or family history of depression (Gawlik *et al*., 2013), young age, low educational level, poor economic-social condition (Bowen & Muhajarine, 2006; Lupattelli *et al*., 2014) smoking cigarettes, a history of premenstrual syndrome (Mei-Dan *et al*., 2015), number of previous pregnancies, unwanted pregnancy, history of abortion, mother’s high level of stress (Ban *et al*., 2014), occurrence of stressful events, lack of family support, lack of economic and emotional support from the husband, fear of delivery, losing their charm for their husbands, and having mixed feelings about taking care of the infant are effective on the occurrence of depression during pregnancy (Johansen *et al*., 2015). Antenatal depression is related to several pregnancy complications that range from relatively mild to more severe. These complications include impaired memory and concentration, weight loss (Bunevicius *et al*., 2009), appetite loss, having negative feelings about oneself, feeling guilty, being disappointed and thinking about hurting oneself (Almeida *et al*., 2016); feeling upset and having low self-esteem, depressed mood, reduced energy and sleep disorders; and not diagnosing and treating depression during pregnancy could lead to undesirable maternal and neonatal outcomes (Malm *et al*., 2015).

Andersson *et al*. (2004) conducted a large, prospective study with 1,495 participants; those with antenatal depression were twice as likely to have nausea, vomiting, and take sick time off work during their pregnancy. They were 1.5 times more likely to visit their obstetrician, compared to participants without depression; and 2.4 times more likely to visit their doctor due to excessive fear of childbirth. Women with antenatal depression also report an increase in somatic symptoms such as headaches, dizziness, difficulty breathing, and stomach pain. Furthermore, Ghaedrahmati et al. (2017) showed that about 50% of women who experience depression during pregnancy would experience post-partum depression too. Thus, attention to depression due to its undesirable impact on the mother, the fetus and the infant is of great importance (Bunevicius *et al*., 2009). Despite this importance, limited research has been conducted to evaluate the pregnancy and neonatal outcomes in women who had depression during pregnancy.

The aim of the present study was to evaluate the relationship between antenatal depression, pregnancy and neonatal outcomes.

## MATERIALS AND METHODS

This cohort study was conducted on 394 pregnant women who were referred to the prenatal care clinic at Al- Zahra Hospital, affiliated to Guilan University of Medical Sciences. Sampling was conducted through convenient (simple) method until the intended sample size was reached.

Considering a 10% possibility of sample loss due to the unwillingness to continue the study, the sample size was increased to 405 participants. From these selected samples, 11 were excluded due to relocation and unwillingness to continue the study. Study enrolment was completely voluntary. The inclusion criteria were 15 to 45 years old, being Iranian, having a singleton pregnancy, having a gestational age of 20 to 28 weeks, not using any drugs, and not having a history of mental illnesses or known depression at any other periods of life. The exclusion criteria were having a history of preterm delivery, having a history of abortion, stillbirths or infertility, having any diagnosed complications during the pregnancy, such as an incompetent cervix, history of having any cardiovascular, respiratory, hematologic, renal, liver, infectious and endocrine diseases, and history of diabetes or hypertension. Data gathering tools were a demographic and obstetric characteristics questionnaire (including age, job, educational status...), the CESD (Center for Epidemiologic Studies Depression) scale for evaluation of depression and a checklist for recording pregnancy outcomes.

Radloff (1977), at the epidemiological research center of the National Institute of Mental Health, has developed a widely used questionnaire for evaluating the epidemiology of depression symptoms in the general population. This questionnaire included 20 questions based on the Likert scale, used to evaluate the symptoms of depression during the week before. Each of the questions would describe one of the symptoms of depression. The symptoms are divided into four classes of mood, intellectual, motivational and physical. Zero to three scores would be assigned to each question in a way that every question except for questions number 4, 8, 12 and 16 would get a 0 for never (less than a day), 1 for a little (1 to 2 days), 2 for sometimes (3 to 4 days) and 3 for a lot (5 to 7 days); but questions number 4, 8, 12 and 16 are positive and would be scored in reverse. The range of scores is from 0 to 60 and higher scores indicate more depression. Based on this tool, a score of less than 15 would be considered as no depression, 15 to 21 as low to moderate depression and higher than 21 is severe depression. The validity of this questionnaire was reported to be 0.85. According to Cronbach’s α, the reliability coefficient of the depression questionnaire used in this study was 0.93. According to the study by Amiri *et al*. (2008) that was conducted in Iran, the reliability of this questionnaire was approved with a Cronbach’s α of 0.92 and its validity was approved using content validity.

The validity of the demographic, social and obstetric characteristics questionnaire was approved using content validity; meaning that the primary version of the questionnaire was developed after reviewing the most recent books and articles about the subject, and after modification, it was distributed among 10 academic members of the Guilan University of Medical Sciences. Then applying their opinions and recommendations, the final version of the questionnaire was developed.

The examiners, who were two midwives working at Al-Zahra hospital of Rasht, were trained to similarly measure the weight, height and head circumference of the infants before the sampling started. After selecting eligible pregnant women, and completely explaining the aims of the study to them, written informed consent was obtained from all the participants. Also, the participants were ensured that they could leave the study at any desired time without any changes to their prenatal care. The participants filled the demographic and obstetric characteristics questionnaire. All the participants were followed-up until the end of their pregnancy to determine their pregnancy and neonatal outcomes. We saved the phone numbers of the mothers and one of her close family relatives. The researchers’ assistant was present at the time of delivery to record the time of delivery, observe, and record the pregnancy and neonatal outcomes. To prevent any bias in data collection, the assistants were not aware of the mothers’ depression condition.

Neonatal variables were categorized as follows: low birth weight was determined as a weight of less than 2,500 grams and very low birth weight as a weight lower than 1,500 grams (Cutland *et al*., 2017). Deliveries before the 37^th^ week of the pregnancy were considered preterm deliveries. In addition, early rupture of the membranes and spontaneous rupture of the membrane before the completion of the 37^th^ week of pregnancy were considered as preterm deliveries (Hidalgo-Chicharro *et al*., 2017). Gestational age was calculated based on the first day of the last menstruation period (LMP) and the performed ultrasounds. Fetal growth restriction was determined through an ultrasound. Abruption placentae is a placenta rupture from its implantation place before the delivery. In the present study, the standard scale of SECA made in Germany with an accuracy of 10 grams was used to measure the weights and for measuring the height, head circumference and chest circumference, a Laika non-stretchable meter with accuracy of 10 millimeters was used. Scale and meter are valid tools for measuring weight and height. To evaluate the reliability of the scale, after weighing 10 participants, the scale was controlled and calibrated using a 100-gram control weight. Simultaneous observing by the researcher and another person of the same height was used to evaluate the reliability of the meter and the information form. To describe the characteristics of the studied participants' descriptive statistics, central indexes, distribution (mean and standard deviation), and frequency distribution were used.

To compare the characteristics of pregnant women between the different groups of body mass index, we used the Chi-square test for qualitative variables and if the Chi-square test was not applicable, we used the Fisher’s exact test. For quantitative variables with normal distribution, repeated measures, Student’s t-test and with abnormal distribution, non-parametric Mann-Whitney test was used. Also, evaluate the correlation between depression and anthropometric indices of the neonate; we used the Pearson’s correlation coefficient.

## RESULTS

In this study, we analyzed data from 394 pregnant women, and from this total, 182 (46.19%) did not have depression, 139 (35.28%) had mild depression and 73 (18.52%) had severe depression. The results of the study showed that the mean age of the participants was 24.63±1.17 years old, the highest frequencies belonged to housewives (67.21), diploma educational level (58.24%), being nulliparous (62.17), and wanted pregnancy (79.41). Table 1 shows the characteristics of the pregnant women based their depression intensity.

**Table 1:** The frequency distribution of the demographic characteristics of studied women based on the intensity of their depression

Variable		No depression (n=182)	Mild (n=139)	Severe (n=73)	*p*-value
		Number (%)	Number (%)	Number (%)	
**Age (year)**	Less than 18	9 (4.94)	12 (8.63)	4 (5.47)	0.01
	18-25	87 (47.80)	65 (46.77)	31 (42.46)	
	25-35	63 (34.61)	23 (16.54)	25 (34.24)	
	More than 35	23 (12.63)	39 (28.05)	3 (4.1)	
**Educational level**	Lower than diploma	32 (17.58)	27 (19.42)	9 (12.32)	0.07
	Diploma	106 (58.24)	84 (60.43)	47 (64.38)	
	Higher than diploma	44 (24.17)	28 (20.14)	17 (23.28)	
**Job**	Housewife	134 (73.62)	96 (69.06)	52 (71.23)	0.31
	Employed	48 (26.37)	43 (30.93)	21 (28.76)	
**Number of pregnancies**	Nulliparous	117 (64.28)	82 (58.99)	49 (67.12)	52.8
	Multigravida	65 (35.71)	57 (41)	24 (32.87)	
**Type of pregnancy**	Wanted	141 (77.47)	102 (73.38)	34 (46.57)	0.03
	Unwanted	21 (11.53)	37 (27)	39 (53.42)	

The occurrence of preeclampsia (*p*<0.01), preterm rupture of the membrane (*p*<0.01), preterm delivery (*p*<0.04), caesarean section (*p*<0.02), intrauterine fetal death (*p*<0.01) and intrauterine fetal growth restriction (*p*<0.001) was significantly higher among mothers with severe depression compared to those who had no depression (Table 2).

**Table 2:** The frequency distribution of the pregnancy outcomes based on the mother’s intensity of depression

Variable		No depression (n=182)	Mild (n=139)	Severe (n=73)	*p*-value
		Number (%)	Number (%)	Number (%)	
Preeclampsia	Yes	4 (2.20)	4 (2.78)	9 (12.32)	<0.01
	No	178 (97.8)	135 (97.13)	64 (87.68)	
Pregnancy diabetes	Yes	6 (3.20)	8 (5.76)	5 (6.85)	0.06
	No	176 (96.8)	131 (71.98)	68 (93.15)	
Preterm rupture of the membranes	Yes	5 (2.75)	7 (5.04)	11 (15.06)	<0.01
	No	177 (97.25)	132 (94.96)	62 (84.94)	
Preterm rupture of the placenta	Yes	1 (0.54)	- (-)	1 (1.36)	0.22
	No	181 (99.46)	139 (100)	72 (98.64)	
Preterm delivery	Yes	34 (18.68)	53 (38.12)	31 (42.46)	0.04
	No	148 (81.32)	86 (67.87)	42 (57.54)	
Cesarean section	Yes	69 (37.91)	98 (70.50)	56 (76.72)	0.02
	No	113 (62.08)	41 (29.50)	17 (23.28)	
Intrauterine fetal death	Yes	- (-)	3 (2.16)	3 (4.14)	<0.01
	No	182 (100)	136 (97.84)	70 (95.89)	
Intrauterine fetal growth restriction	Yes	2 (1.10)	1 (0.72)	7 (9.58)	<0.001
	No	180 (98.9)	138 (99.28)	66 (94.42)	

In addition, results from the present study showed that the birth weight of neonates from mothers with severe depression was lower than those of mothers with mild depression, and their difference was statistically significant (*p*<0.001). The mean height and head circumference of the neonates from mothers who had no depression were bigger than those of depressed mothers, but their difference was not significant (Table 3).

**Table 3:** The frequency distribution of the neonates’ characteristics based on the mothers’ intensity of depression

Variable	No depression	Mild	Severe	*p*-value
	Mean ± SD	Mean ± SD	Mean ± SD	
Weight (kg)	3143±1.61	3210±2.16	2815±1.51	<0.001
Height (cm)	50.60±2.45	50.13±3.38	49.91±2.96	0.17
Head circumference (cm)	35.72±2.21	35.17±3.72	34.93±1.45	0.06

In addition, Table 4 shows the neonatal outcomes based on the intensity of their depression.

**Table 4:** Neonatal outcomes based on the intensity of the mother’s depression

Variable		No depression (n=182)	Mild depression (n=139)	Severe Depression (n=73)	*p*-value
Birth weight	≥2500	159 (87.36)	104 (74.82)	51 (69.86)	<0.0001
	<2500	23 (12.63)	28 (20.14)	22 (30.13)	
Infant’s Apgar score 1 minute after birth	≥7	178 (97.81)	133 (95.68)	69 (94.52)	0.09
	<7	4 (2.24)	6 (4.32)	4 (5.48)	
Infant’s Apgar score 5 minutes after birth	≥7	180 (98.97)	133 (95.68)	71 (97.26)	0.12
	<7	2 (1.15)	6 (4.32)	2 (2.74)	
Respiratory distress at birth	Yes	4 (2.24)	3 (2.15)	2 (2.74)	0.36
	No	178 (97.81)	136 (97.84)	71 (97.26)	
Abnormality	Yes	1 (0.54)	- (-)	- (-)	0.59
	No	181 (99.46)	139 (100)	73 (100)	

## DISCUSSION

This study clearly reveals a strong relationship between antenatal depression symptoms and pregnancy and neonatal outcomes. 18.52% of the pregnant women had severe depression. Moshki & Cheravi (2016) reported 37% of women who met the criteria for a depressive disorder during pregnancy. However, comparison of study results of both prevalence and incidence is complicated due to the use of different screening/diagnostic tools, different cutoff points to determine postpartum depression, varying time points in pregnancy and postpartum when the symptoms are assessed, and cross-cultural variables. Some studies on depressed pregnant women have reported negative neonatal outcomes such as preterm delivery, low birth weight and fetal growth restriction, which are similar to the results of the present study (Accortt *et al*., 2015; Loomans *et al*., 2013; Liu *et al*., 2016). Preeclampsia had also been increased in depressed participants. Avalos *et al*. (2015) reported that preeclampsia was one of the outcomes that was significantly higher among depressed mothers compared to mothers with no depression. This high risk is due to the increased blood pressure following depression. Furthermore, Hu *et al*. (2015) found that participants with depression in the second trimester were significantly more likely to have preeclampsia. Preterm rupture of the membrane among depressed mothers was higher compared to mothers with no depression. In this regard, the results of the present study were in contrast with the results from Accortt *et al*. (2015). The reason for this difference could be due to the small sample size from Accortt *et al*. Preterm delivery was higher among depressed participants than participants without depression were. Mochache *et al*. (2018) reported participants were nearly more likely to have induced labor if they had antenatal depression continuously throughout pregnancy.

In our study, most of the mothers who did not have depression had a vaginal delivery, while most of the mothers with severe depression were submitted to a cesarean section. Chang *et al*. (2014) found that participants with depression in the third trimester were more likely to have an epidural and caesarean birth. This study reveals that women with antenatal depression exhibit an increased likelihood of giving birth to LBW infants. This is consistent with previous studies from other South Asian countries documenting that women who exhibit elevated depressive symptoms during pregnancy are at increased risk of delivering LBW infants (Chaman *et al*., 2013). Results from high-income countries have found a similar association, but only in the socially deprived group (Şahingöz *et al*., 2014). It may be due to etiological heterogeneity across these settings, because of the different cultures, health-care systems, and maternal and child health profiles. Rahman *et al*. explored complications in 143 depressed mothers compared to 147 non-depressed mothers and found that participants experiencing depression in the third trimester had infants born with significantly lower birth weights when compared to non-depressed participants (Rahman *et al*., 2007).

## CONCLUSION

This study demonstrates that major depression and mild depression can have a negative impact on pregnancy and delivery outcomes. While there were less significant associations than expected, and no significant associations with neonatal outcomes, this study complements the existing literature by demonstrating in what stage of pregnancy depression may have an impact on the outcomes.

This study had several strengths, including the comprehensive questionnaires completed by the participants, the use of the EPDS as a valid and reliable screening tool, and the fact that it was a prospective, cohort study. This study clearly demonstrates that both mild and major depression can seriously affect pregnancy and delivery outcomes. This study has two important potential implications. Firstly, given the frequency of depression during pregnancy, healthcare professionals need to be aware of the potential health risks of both conditions, to both the mother and the baby. With increased awareness of these associations, healthcare professionals may also become more cognizant about the presence of antenatal depression in their patients and become more likely to encourage women to seek treatment for these issues. Secondly, it is important that women are aware of possible complications and know that depression is common during pregnancy. Limitations of this study were that EPDS are self-report measures, which may be subject to response bias. Due to the use of self-report measures, participants’ responses were restricted, which meant that the elaboration of answers was not possible. The small sample size that resulted may have decreased statistical power, indeed, multiple regression analyses would be worthy of consideration for future analyses.
